# P43 A complicated road to diagnosis: Auto inflammatory bone disease

**DOI:** 10.1093/rap/rkac067.043

**Published:** 2022-09-28

**Authors:** Indhuja Rajarathinam, Vinay Shivamurthy

**Affiliations:** Guy's and St Thomas', London, United Kingdom; Guy's and St Thomas', London, United Kingdom

## Abstract

**Introduction/Background:**

Auto inflammatory bone disorders are characterized by chronic non-infectious osteomyelitis and inflammation-induced bone resorption. It results from aberrant activation of components of the innate immune system. The auto inflammatory bone disorder includes chronic non-bacterial osteomyelitis (CNO) and its severe form chronic recurrent multifocal osteomyelitis (CRMO); synovitis, acne, pustulosis, hyperostosis, osteitis (SAPHO) syndrome; Majeed syndrome; deficiency of interleukin-1 receptor antagonist (DIRA) and cherubism. The most common among this is CRMO/CNO. The disease can affect any part of the body but the metaphyseal region of the long bones, clavicle, and vertebra are the most commonly affected site.

**Description/Method:**

A 9 year old girl presented with five month history of swelling over right periocular and temporal region, associated with skin discoloration and difficulty in opening her eyes. She also reported headache more in the right frontal region. She had on and off exacerbations of skin discoloration and headaches. There was no history of fevers, weight loss, night sweats, skin lesions or arthritic symptoms. She did not have a personal or family history of inflammatory or autoimmune disorders. She is under ophthalmology elsewhere since the age of 3 months with a right divergent squint and history of right squint surgery at 6 years.

Physical examination revealed swelling involving right infraorbital and temporal region, which was soft and not tender to touch with associated erythema in the same site. There were no signs of meningism or altered mental status. Musculoskeletal examination was unremarkable.

Her blood test results showed mild increase in platelets (476 * 109), CRP 8mg/L, ESR (18mm/hr). IgG4 titres were mildly raised (1.16g/L). Other immunologic markers including rheumatoid factor, human leukocyte antigen B27, antinuclear antibody and anti-neutrophil cytoplasmic antibody were normal.

Histopathological examination of bone and soft tissue of her right orbital rim showed low grade non-specific chronic inflammation with extensive appositional remodelling of bone suggestive of CRMO/SAPHO. No evidence of malignancy/infection.

Later whole body MRI was performed to identify the extent of disease. MRI whole body was performed which showed multiple areas of bone marrow oedema and periostitis within the appendicular and axial skeleton, bilateral gluteal enthesitis and sacroilitis. There are some features that are atypical for CRMO (asymmetrical involvement, transphyseal involvement including epiphyseal involvement of the left humerus). Also noted to have right-sided periorbital oedema, oedema of the right masticator muscles and temporalis. Further planned to do dedicated MRI of left humerus with contrast.

**Discussion/Results:**

It can be difficult to consider what the problem is when a health care provider is presented with a group of seemingly disparate signs and symptoms with a history and time course that do not match classic (or commonly atypical) disease presentations. Common things happen commonly, so when symptoms occur without the usual co-occurring symptoms, unusual symptoms or time courses, it can be challenging.

The current patient presented with periocular swelling with headache and skin discolouration. The differentials can be Langerhans cell histiocytosis, IgG4-related disease, osteoblastoma, and osteosarcoma. There were no hepatosplenomegaly, lymphadenopathy, also the mass was soft to palpate, and hence the above differentials were unlikely. Patient was initially evaluated at tertiary oncology centre and malignancy ruled out. The histopathology slides were reviewed and reported as mentioned above. In view of no skin manifestations, CRMO was the likely diagnosis considered. Hence MRI whole body performed.

Barrani et al reported a similar type of unusual case of a 12-year-old girl with CRMO arising with recurrent episodes of left supraorbital headache, followed by the appearance of a periorbital dyschromia. Magnetic resonance imaging (MRI) of the skull and orbits revealed an important subacute inflammatory process. However, a few months later, the child presented a painful swelling of the left clavicle; the histological examination of the related biopsy allowed us to establish the diagnosis of CRMO.

The follow up with our patient is quite a short duration and she might evolve later in the disease course giving more clarity!

**Key learning points/Conclusion:**

In evaluating a diagnostic dilemma, starting the laboratory evaluation helps to begin the process of trying to find an answer and can also help the families as “something is being done.”

Chronic recurrent multifocal osteomyelitis can present with unifocal lesions, atypical locations or absence of recurrence.

A high level of suspicion is paramount to avoid unnecessary biopsies and repeated antibiotic regimens.

Families will often be quite concerned and support for them is also necessary and close communication and follow up is appropriate.

